# Electrical Resistivity-Based Study of Self-Sensing Properties for Shape Memory Alloy-Actuated Artificial Muscle

**DOI:** 10.3390/s131012958

**Published:** 2013-09-26

**Authors:** Jian-Jun Zhang, Yue-Hong Yin, Jian-Ying Zhu

**Affiliations:** State Key Laboratory of Mechanism System and Vibration, Institute of Robotics, Shanghai Jiao Tong University, Shanghai 200240, China; E-Mails: zhangjianjun@sjtu.edu.cn (J.-J.Z.); zjyao@nuaa.edu.cn (J.-Y.Z.)

**Keywords:** shape memory alloy, artificial muscle, self-sensing model, electrical resistivity, active ankle-foot orthosis

## Abstract

Shape memory alloy (SMA) has great potential to develop light and compact artificial muscle (AM) due to its muscle-like high power-to-weight ratio, flexibility and silent operation properties. In this paper, SMA self-sensing properties are explored and modeled in depth to imitate the integrated muscle-like functions of actuating and self-sensing for SMA-AM based on the investigation of SMA electrical resistivity (ER). Firstly, an ER transformation kinetics model is proposed based on the simulation of SMA differential scanning calorimetry (DSC) curves. Then a series of thermal-electrical-mechanical experiments are carried out to verify the validity of the ER model, whereby the SMA-AM self-sensing function is well established under different stress conditions. Finally the self-sensing capability is further demonstrated by its application to a novel SMA-AM-actuated active ankle-foot orthosis (AAFO).

## 1. Introduction

Shape memory alloy (SMA) actuators, which have muscle-like functions of actuating, structure and high power-to-weight ratio, are becoming increasingly popular for artificial muscle (AM) design [1–3]. Compared with traditional actuator-based AM, such as DC motor [4–6] and pneumatic AM [7], SMA-based AM not only can be used for actuating, such as in artificial hands [8,9] or bipeds [10], but also can be used for self-sensing, such as the flexures designed by Lan *et al.* [11,12] and the AM developed by Zhang *et al.* [2]. For decades, many scholars have conducted and are still conducting research on SMA self-sensing properties in order to imitate the integrated muscle-like functions of actuating and self-sensing for SMA-AM [2,11–14]. The self-sensing function mainly refers to using SMA resistance as the feedback signal for SMA length. It has been recognized that resistance plays a significant role in SMA self-sensing utilization, while there has been little research that explores and develops the self-sensing models due to the complexity of electrical resistivity (ER) variations. SMA mainly has two phases, namely martensite (M) and austenite (A). When being heated, the SMA changes its shape from M to A. Subsequent cooling returns its shape from A to M under stress [15]. For a certain given SMA wire, the intermediate R-phase (R) will also appear between the transformation from A to M during cooling. The ER is not only dependent on the stress and temperature, but also on the volume fractions of M, A and R during transformation [16]. Furthermore, the intermediate R-phase has different ER properties comparing with M and A, which further increases the complexity of the self-sensing modeling.

Up to now, few self-sensing models have been proposed in the literature [2,11,14]. Lan *et al.* [11] developed a self-sensing model directly based on the SMA strain to resistance curves by polynomial fitting without considering the ER effect. Novák *et al.* [14] proposed a micromechanical model for ER variations based on s detailed analysis of the factors influencing ER variations from a microscopic perspective, such as stress, temperature, martensite fraction, martensite texture and R-phase distortion. Reasonable agreement between the simulation and experimental result of resistivity responses was achieved. However, apart from stress and temperature, the other three factors as mentioned above are difficult to measure by real-time control methods, which is not conducive to feedback control applications. In addition, these three factors are closely related to stress and temperature, and the ER variations can actually be attributed to the direct and indirect effect of stress and/or temperature. Besides, though R-phase may complicate the ER variations, the ER curves of *A*→*M* transformation and *A*→*R* transformation have good similarity according to our experimental results. Furthermore, similar to the linearity between resistance and strain during *A*– *M* forward and reverse transformation, the resistance and strain also has good linearity during *A*– *R* forward and reverse transformation [16]. Thus it is probable to develop more practical and accurate self-sensing model based on ER variations from control perspective.

The motivation of this paper was to propose a self-sensing model for SMA-actuated AM to establish resistance to length (R-L) relation based on deeply exploring inherent ER properties of SMA wires. The SMA-AM presents integrated muscle-like functions of actuating and self-sensing. Finally, we propose a novel active ankle-foot orthosis (AAFO) actuated and sensed by the SMA-AM to demonstrate the self-sensing capability. Experimental results show that the SMA-AM is robust to external stress variations and accurate angle control using is achieved the self-sensing method.

## 2. Analysis of ER Properties of SMA Actuated AM

**Figure 1.**
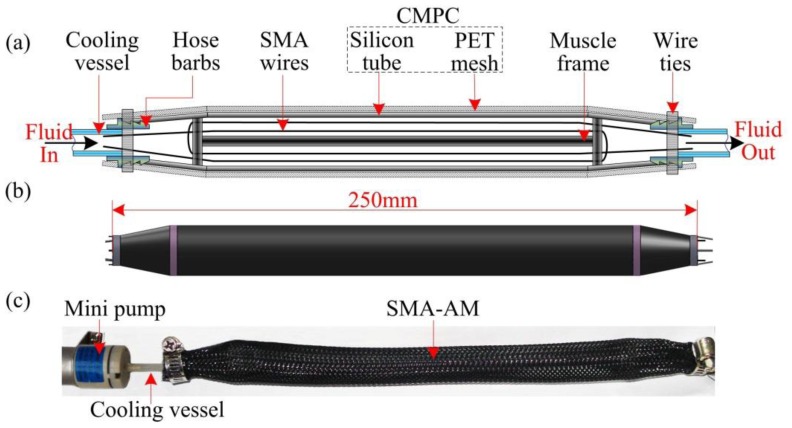
Schematic (**a**), CAD model (**b**) and picture (**c**) of the SMA-AM structure.

SMA wire can be used as the active contractile element for artificial muscle design due to its muscle-like properties. The designed SMA-actuated AM, as shown in Figure 1, presents multifunctional actuating, energy-storing and self-sensing properties [1,2]. To obtain an accurate self-sensing function, the resistance-length (R-L) relationship should be correctly established. However, the R-L relationship has not yet been entirely explored due to the complexity of electrical resistivity (ER) variations.

**Figure 2.**
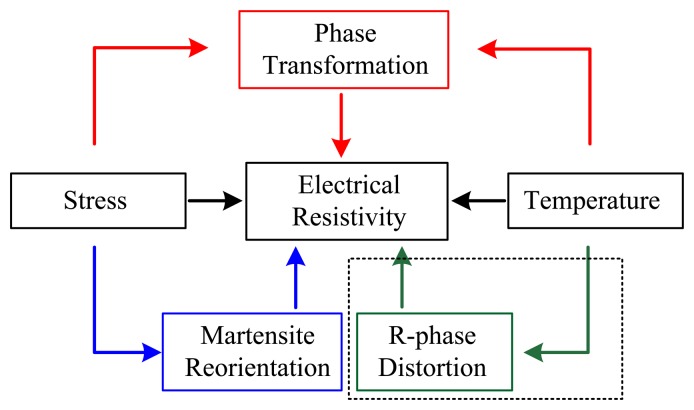
Relationship of influencing factors on ER variation of SMA wires.

For a certain given SMA wire, ER variations not only depend on the direct effects of stress and temperature, but also on stress and/or temperature induced phase transformation, martensite reorientation and R-phase distortion. The detailed relationships of the influencing factors on ER variations are shown in Figure 2. As with common metals, the ER of SMA changes linearly with temperature and stress at stable individual phases (black arrow) since SMA is a metal. However, the ER is also closely related to phase transformation induced by stress and/or temperature (red arrow), and martensite reorientation induced by applied stress (blue arrow), which is in contrast to the behavior of common metals. Besides, when the R-phase appears in SMA, the ER also changes linearly with R-phase distortion induced by temperature (green arrow). Consequently, ER variations are attributed to the direct and indirect effect of stress and/or temperature for a certain given SMA wire. In this study, equiatomic NiTi wires were investigated as the sample obtained from Dynalloy, Inc. (Tustin, CA, USA).

When no transformation occurs, the temperature coefficient of resistivity (TCR) and stress coefficient of resistivity (SCR) can be used to denote the direct effect of temperature and stress on the ER. Furthermore, martensite reorientation is proportional to applied stress and R-phase distortion is inversely proportional to temperature [14]. Thus, positive SCR and negative TCR can be used to denote the effect of martensite reorientation and R-phase distortion on ER respectively, and all the coefficients gained from experiments are listed in Tables 1 and 2. Therefore, the following linear equation can be obtained to express ER variations of individual phases outside the phase transformation region:

(1) $$\rho_{iT}^{\sigma }=\rho_{0i}^{0}+\alpha_{i}(T-T_{0i})+\beta_{i}\sigma$$

where subscript *i* represents A, M or R, *T* is temperature and σ is applied stress on SMA wire. $p_{0i}^{0}$ is ER of individual phases measured at temperature *T*_0_*_i_* and zero stress. α*_i_* is TCR and *β_i_* is SCR and $p_{iT}^{\sigma }$ is ER of individual phases at temperature *T* and stress σ.

During phase transformation, ER variations depend mainly on the volume fractions of austenite and martensite phases, which in turn depend on the stress and/or temperature. The appearance of R-phase in SMA wire complicates the change of ER, but we find that the ER variations of *A*→*M* and *A*→*R* have good similarity. Thus we mainly focus on the study of ER variations of phase transformation without R-phase, which can be deduced reasonably to the situation with R-phase.

## 3. DSC Curve Analysis and ER Modeling

In order to study ER variations during transformation, DSC tests were performed to analyze the phase transformation of SMA wire under zero stress by employing a Netzsch DSC 200 F3 Maia^®^ Instrument (Burlington. MA, USA). Three identical thermal cycles were performed on the sample. For each cycle, the temperature cycle range was from −10 °C to 120 °C at a constant heating and cooling rate of *dT*/*dt* = 10 °C/min.

**Figure 3.**
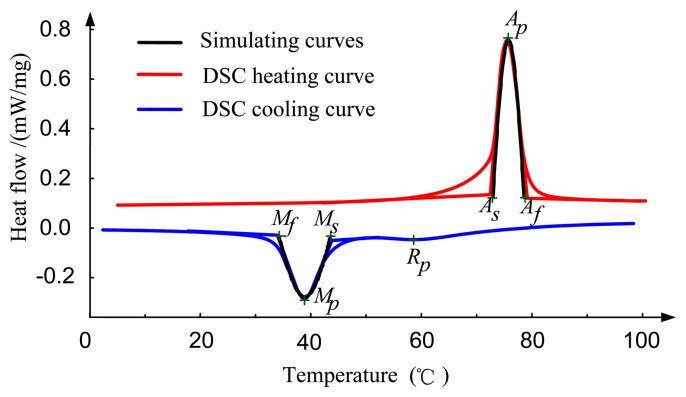
Measured and simulated DSC curves of SMA wires.

Figure 3 shows the third cycle heat flow *vs.* temperature curves of the SMA wires. An endothermic and two exothermic peaks (one exothermic peak is apparent while the other is not) are present due to latent heat absorption and release. *M_s_*, *M_p_* and *M_f_* are martensite start, peak and finish temperature respectively during cooling. *A_s_*, *A_p_* and *A_f_* are austenite start, peak and finish temperature, respectively, during heating. *R_p_* is R-phase peak temperature, while R-phase start *R_s_* and finish temperature *R_f_* are not detectable by DSC. Here we temporarily ignore the impact of R-phase. Note that the temperature parameters mentioned above approximately satisfy the following relationships:

(2) $$A_{p}=\frac{A_{f}+A_{s}}{2},M_{p}=\frac{M_{f}+M_{s}}{2}$$

In order to clarify thermal transformation behavior of SMA, we use a “Cosine curve” to simulate relationship between heat flow and temperature during transformation as shown in Figure 3 (black line). This approach relies on the notion that the gradual phase transformation may be represented by relatively symmetric DSC peaks. Here reverse transformation is taken as a case study and the expression for heat flow and temperature is given as follows:

(3) $$f(T)=h\cos[a_{A}(T-A_{p})]+C_{1}$$

where *T* is temperature of SMA, *h* and *C*_1_ are constants and *a_A_*= π/(*A_f_* − *A_s_*).

Furthermore, latent heat *Q* associated with phase transformation can be calculated by integrating Equation (3) between *A_s_* and *A_f_*. They satisfy the following expression:

(4) $$\frac{dQ}{dT}=f(T)\frac{dt}{dT},A_{s}<T<A_{f}$$

According to the analysis in the last section, the ER variations of SMA wires depend mainly on the volume fractions of austenite and martensite phases during transformation, which actually depend on the extent of phase transformation. In addition, the gradual change of latent heat absorption and release can be used to represent the extent of phase transformation [17], thus the following equation can be obtained:

(5) $$\frac{d\rho_{M\to A}}{dT}=k\frac{dQ}{dT}+C_{2}$$

where *k* and *C*_2_ are constants. Substituting Equations (3) and (4) into Equation (5) leads to:

(6) $$\frac{d\rho_{M\to A}}{dT}=H\cos[a_{A}(T-A_{p})]+C$$

where *C* = *kC*_1_ + *C*_2_and *H* are constants. When *T* = *A_s_* or *A_f_*,*d ρ*_M→_*_A_*_/_*_dT_*_= 0_. Integrating Equation (6), we can obtain the following relationship between ER and temperature during reverse transformation:

(7) $$\begin{array}{c} \rho_{M\to A}=\frac{\rho_{A_{f}}^{0}-\rho_{A_{s}}^{0}}{2}\sin[a_{A}(T-A_{p})]+\frac{\rho_{A_{f}}^{0}+\rho_{A_{s}}^{0}}{2}\\ \text{for}A_{s}\leq T\leq A_{f} \end{array}$$

where $\rho_{A_{s}}^{0}$ and $\rho_{A_{f}}^{0}$ are the start and end ER of reverse transformation at zero stress, which can be calculated by Equation (1). Likewise, we can obtain ER *vs.* temperature during forward transformation:

(8) $$\begin{array}{c} \rho_{A\to M}=\frac{\rho_{M_{s}}^{0}-\rho_{M_{f}}^{0}}{2}\sin[a_{M}(T-M_{p})]+\frac{\rho_{M_{s}}^{0}+\rho_{M_{f}}^{0}}{2}\\ \text{for}M_{f}\leq T\leq M_{s} \end{array}$$

where $\rho_{M_{s}}^{0}$ and $\rho_{M_{f}}^{0}$ are the start and end ER of forward transformation at zero stress, which can be calculated by Equation (1). Besides, *a_M_*= π/(*M_s_* − *M_f_*)

When stress is applied to SMA during thermal cycle, SMA transformation temperature will vary linearly [18], and the ER will also vary correspondingly. Besides, the ER varies linearly with applied stress according to our experimental results. Similar results are shown in literature [19]. In order to correctly model the relationship of ER, temperature and applied stress during reverse transformation, the following expression is proposed:

(9) $$\begin{array}{c} \rho_{M\to A}=\frac{\rho_{A_{f}}^{0}-\rho_{A_{s}}^{0}}{2}\sin[a_{A}(T-A_{p})+b_{A}\sigma ]+\frac{\rho_{A_{f}}^{0}+\rho_{A_{s}}^{0}}{2}+\beta_{A}\sigma \\ \text{for}\;A_{s}\leq T\leq A_{f} \end{array}$$

where *b_A_*= −*a_A_*/*C_A_* and *C_A_* indicates the influence of stress on reverse transformation temperatures. *β_A_* is SCR of austenite. Likewise, we can obtain ER, temperature and applied stress relationship during forward transformation:

(10) $$\begin{array}{c} \rho_{A\to M}=\frac{\rho_{M_{s}}^{0}-\rho_{M_{f}}^{0}}{2}\sin[a_{M}(T-M_{p})+b_{M}\sigma ]+\frac{\rho_{M_{s}}^{0}+\rho_{M_{f}}^{0}}{2}+\beta_{M}\sigma \\ \text{for}\;M_{f}\leq T\leq M_{s} \end{array}$$

where *b_M_*= −*a_M_*/*C_M_* and *C_M_* indicates the influence of stress on forward transformation temperatures. *β_M_* is the SCR of martensite. When R-phase appears, forward transformation will be separated into two steps (*A*→*R* and *A*→*M*), and the following two expressions can be obtained based on the same analysis of *A*→*M*:

(11) $$\{\begin{array}{l} \rho_{A\to R}=\frac{\rho_{R_{s}}^{0}-\rho_{R_{f}}^{0}}{2}\sin[a_{R}(T-R_{p})+b_{R}\sigma ]+\frac{\rho_{R_{s}}^{0}+\rho_{R_{f}}^{0}}{2}+\beta_{R}\sigma \\ \rho_{R\to M}=\frac{\rho_{M_{s}}^{0}-\rho_{M_{f}}^{0}}{2}\sin[a_{M}(T-M_{p})+b_{M}\sigma ]+\frac{\rho_{M_{s}}^{0}+\rho_{M_{f}}^{0}}{2}+\beta_{M}\sigma \end{array}$$

where $\rho_{R_{s}}^{0}$ and $\rho_{R_{f}}^{0}$ are the start and end ER of *A*→*R* transformation at zero stress, which can also be calculated by Equation (1). Besides,*a_R_* = π/(*R_s_*−*R_f_*), *b_R_* = −*a_R_*/*C_R_* and *C_R_* indicates the influence of stress on *A*→*R* transformation temperatures.

In the next section, we will validate the efficiency and accuracy of the developed model through experiments.

## 4. Experimental Setup

**Figure 4.**
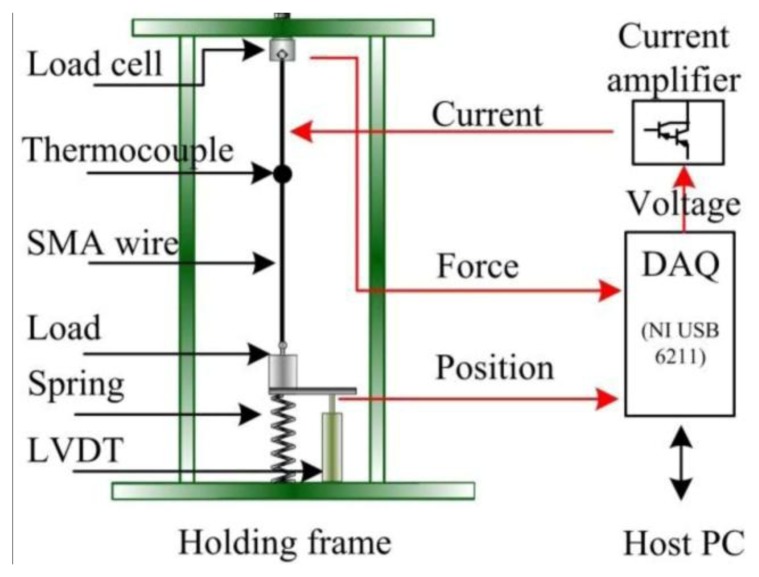
Schematic diagram of the experimental setup.

To study ER characteristics during transformation, we conducted a series of thermal-electrical-mechanical experiments to the as-received SMA sample, as shown in Figure 4. The sample with diameter of 0.15 mm and length of 117 mm is connected to different constant loads varying from 28 to 224 MPa and a helical bias spring with stiffness of 390 N/m. A load cell is connected to the SMA wire to measure the contraction force. A linear variable differential transformer (LVDT) with resolution of 0.01 mm measures the SMA wire length change. Besides, a thermocouple (Omega type K) is positioned at the center of the SMA wire to measure the temperature. The wire is sheltered from air draughts. Heating current generated from current amplifier is used to drive the SMA wire and the resistivity is computed according to the following equation [20]:

(12) $$\rho =R\frac{S}{l}=\frac{U}{I}\frac{V_{0}}{l^{2}}$$

where *U* is the voltage and *I* is the heating current across the SMA wire. *l* is the wire length. *V*_0_ is the wire volume which can be considered as a constant. A computer equipped with a data acquisition card (NI USB-6211) is used to store and process all the measured data.

## 5. Experimental Results and Discussion

### 5.1. Heat Transfer Analysis of SMA Wire

The thermocouple is capable of detecting steady state temperature or slow response temperature, but it is hard to detect fast response temperatures, and it is also hard to detect the temperature changes induced by latent heat absorption/release during fast phase transformation. In addition, with a view towards real-time feedback control applications, it is more practical to control heating/cooling time rather than temperature of SMA wires. Therefore, heat transfer analysis based on lumped parameter method (LPM) and energy conservation principle is performed to investigate the temperature response of SMA wires. Then the temperature *vs.* time and ER *vs.* time relationship can be established.

The heat transfer models outside phase transformation and during phase transformation are written as Equations (13a) and (13b) respectively:

(13a) $$mC_{V}\frac{dT}{dt}=I^{2}R-hA_{\text{surf}}(T-T_{\text{amb}})$$

(13b) $$m(C_{V}+Q_{i}\frac{d\zeta }{dT})\frac{dT}{dt}=I^{2}R-hA_{\text{surf}}(T-T_{\text{amb}})$$

where *m* is the mass and *A_surf_* is the surface area of SMA wire, *C_v_* is heat capacity, *R* is the average resistance of SMA wire. *T_amb_* is the reference temperature, taken to be identical to the ambient temperature of the environment, *I* is the electric current, and *h* is the convection coefficient. Note that radiative effects are lumped into the convective term. *Q_i_* is the latent heat associated with the phase transformation. The subscript *i* refers to *M*→*A* during heating and *A*→*M* during cooling. Latent heat release of *A*→*R* transformation is ignored because no obvious heat flow occurs, as shown in Figure 3. In addition, ξ refers to martensitic volume fraction, which may be taken to be solely dependent on temperature for purely thermal transformation [21], and the specific functional dependence can follow from a simulation of DSC curves of the heat flow *vs.* temperature for the SMA wires. Considering Equation (3) and the critical value of ξ (When *T* = *A_S_*, ξ = 1 and *d*ξ/*dT* = 0), we can gain the following expressions:

(14a) $$\frac{d\xi }{dT}=-\frac{a_{A}}{2}\cos[a_{A}(T-A_{p})]\;\text{for}\;M\to A$$

(14b) $$\frac{d\xi }{dT}=-\frac{a_{M}}{2}\cos[a_{M}(T-M_{p})]\;\text{for}\;A\to M$$

**Figure 5.**
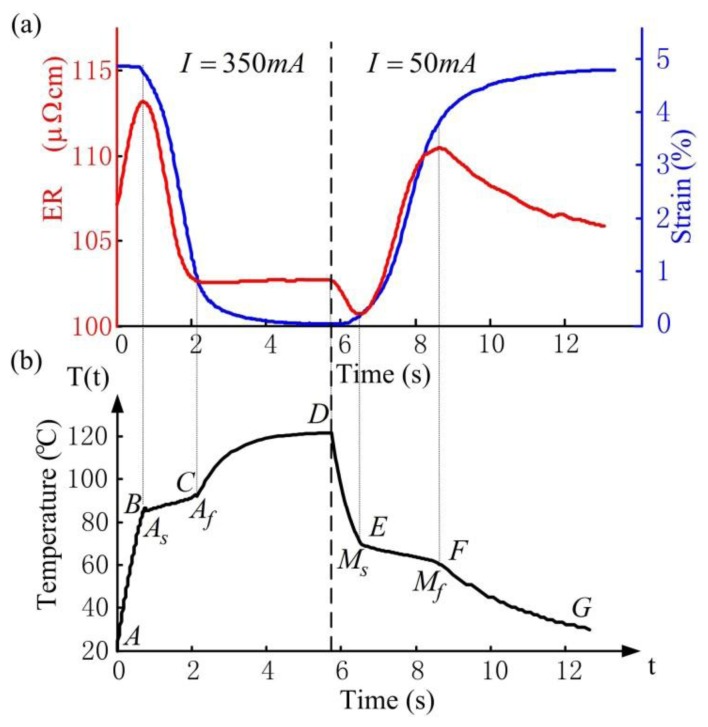
Experimental ER and strain response (**a**) and numerical temperature response (**b**) of SMA wire during heating (I = 350 mA) and cooling (I = 50 mA).

The thermal response is obtained by numerically integrating the above Equations (13a)–(14b), and the temperature *vs.* time curve during heating (*I* = *350 mA*) and cooling (*I* = *50 mA*) is shown in Figure 5b. Note that outside the transformation region, the temperature changes in exponential form (segments AB, CD and segments DE, FG), while during transformation the latent heat absorption and release cause the sudden change of the slope, as is shown in segments BC and EF, which denote *M*→*A* and *A*→*M* transformation, respectively. The simulation results in Figure 5b are consistent with experimental results in literature [21]. For simplicity, temperature *vs.* time relationship can be approximately considered linear during transformation according to the simulation results, and the following temperature *vs.* time functions are obtained:

(15a) $$T=K_{c}e^{t/\tau }+T_{c}\text{for non}‐\text{transformation}$$

(15b) $$T=K_{t}t+T_{s}\text{for transformation}$$

where *K_C_* = *T*_0_−*T_amb_* −*I*^2^*R*/*hA_surf_*, *T_C_* =*T_amb_* +*I*^2^*R*/*hA_surf_* and τ = −*mC_V_*/*hA_surf._T*_0_ is the start temperature of heating/cooling, *T_s_* is the start temperature of transformation and *K*_t_ is the slope of BC and EF.

The corresponding experimental results of ER (red line) and strain (blue line) response of the SMA wire are shown in Figure 5a. During heating, the ER response is non-monotonic with a rapid increase, decrease then increase again. In segment BC, the SMA wire undergoes reverse transformation, where the ER decreases because the ER of austenite is smaller than that of martensite. In segments AB and CD, no transformation occurs, where the ER increase is due to heating. In addition, the strain changes slightly in segments AB and CD but decreases rapidly in segment BC due to reverse transformation. During cooling, the opposite characteristics of ER and strain response occur. In segment EF, the ER increases due to forward transformation while in segments DE and FG, the ER decrease is due to cooling. Churchill *et al.* [22] showed similar results to those presented in Figure 5a through an external heating method.

### 5.2. Experimental Results at Various Constant Stresses

**Figure 6.**
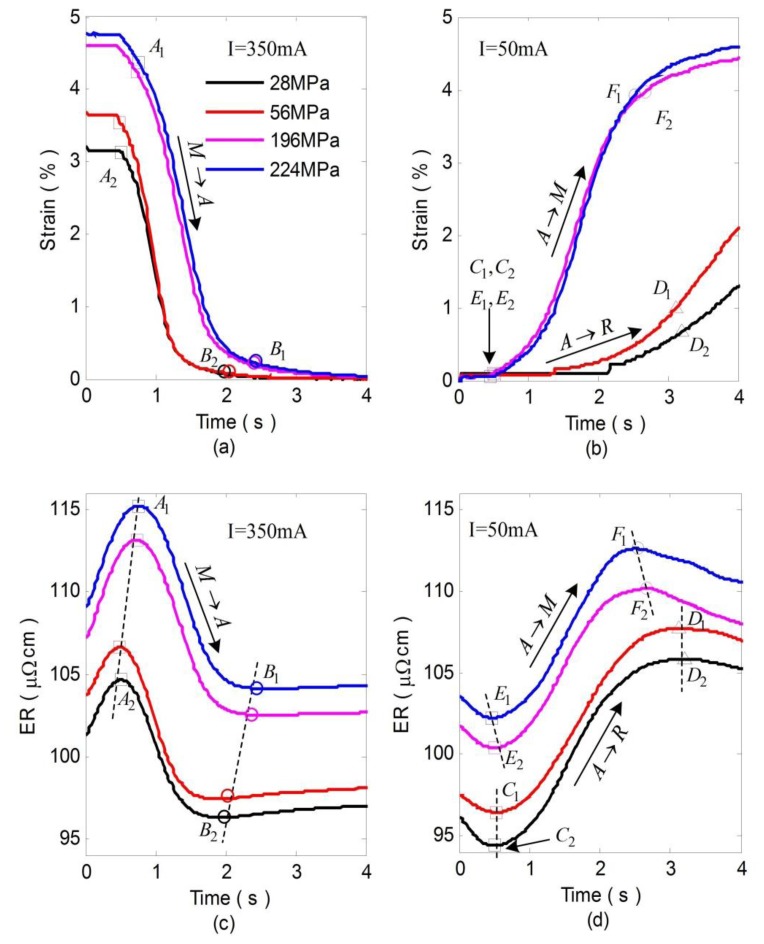
Experimental results of strain (**a**,**b**) and corresponding ER (**c**,**d**) changes of SMA wire during heating (I = 350 mA) and cooling (I = 50 mA) at various stresses.

**Figure 7.**
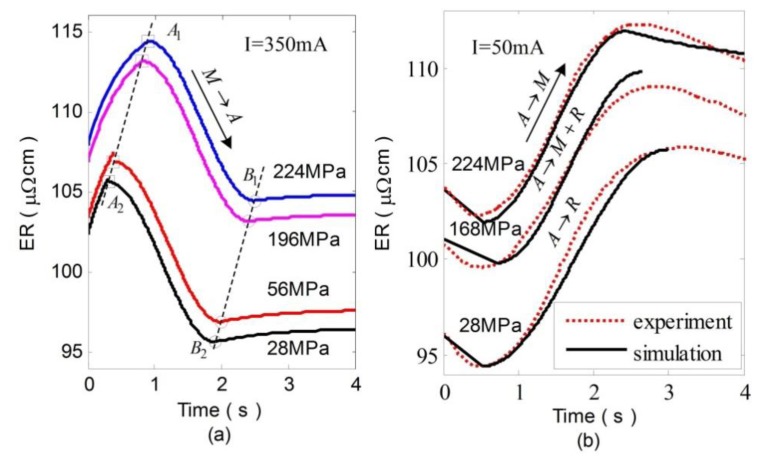
Simulation of ER changes of SMA during heating (**a**) and cooling (**b**) at various stresses.

Figure 6a,c show the experimental results of strain and corresponding ER curves during heating at various constant stresses. With the increase of the stress, the start (denoted by *A*_1_*A*_2_) and end (denoted by *B*_1_*B*_2_) temperature of the reverse transformation increase correspondingly. In addition, the whole ER value also increases in proportion to the stress. Besides, the simulation results of ER during heating can be obtained by combining Equations (1) and (9) with Equation (15), as shown in Figure 7a. The simulations agree reasonably well with the experimental results. Figure 6b,d shows the strain and corresponding ER curves during cooling at various constant applied stresses. Comparing with Figure 6a, the strain curves shown in Figure 6b are quite different as stress varies, which is caused by R-phase [17]. In contrast to the *A*→*M* transformation which yields a large shape change (5%), the *A*→*R* transformation yields only a small shape change (less than 1%).

During cooling, As is shown in Figure 6d, the ER increase mainly denotes *A*→*R* transformation (from *C*_1_*C*_2_ to *D*_1_*D*_1_) and *A*→*M* transformation (from *E*_1_*E*_2_ to *F*_1_*F*_2_) when the stress is lower than 56 MPa and higher than 196 MPa. This is because all the transformation temperatures increase linearly with increasing applied stress, but *R_s_* exhibits a stress dependence substantially smaller than that of *M_s_* or *A_f_* [16], thus R-phase fraction decreases gradually with the increase of the stress. For stresses greater than a certain value, *M_s_* exceeds *R_s_* and only one step (*A*→*M*) appears during the forward transformation.

**Figure 8.**
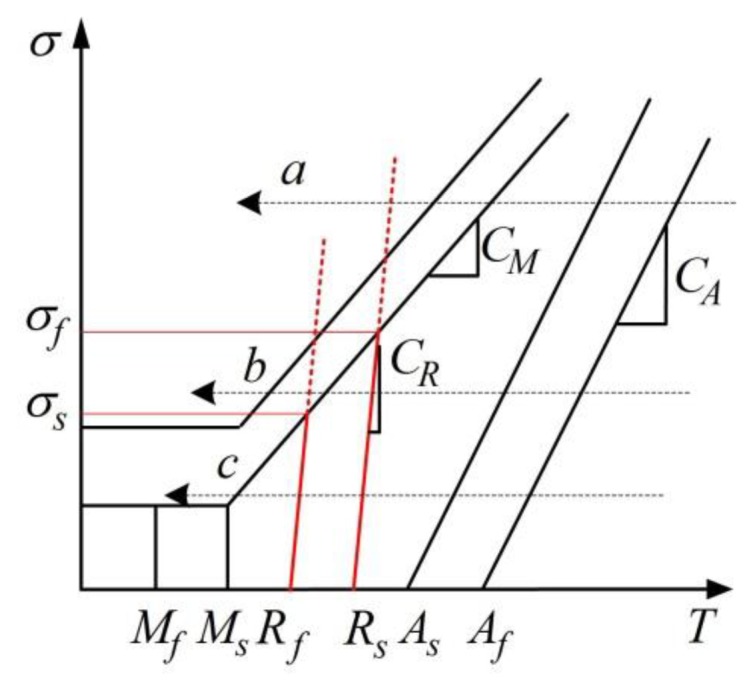
Revised critical stress-transformation temperature relationship.

A revised critical stress-temperature plot including R-phase is shown schematically in Figure 8 to illustrate this situation. If applied stress is higher than *σ_f_*, no R-phase occurs during cooling (arrow a). If stress is lower than *σ_s_*, two separate steps (*A*→*R*→*M*) appear successively (arrow c). If stress is within the above range, two mixed steps (*A*→*R*+*M*) appear (arrow b). Here, three constant applied stresses (28 MPa, 168 MPa and 224 MPa) are implemented as case studies.

Combining Equations (1), (10), (11) with Equations (15), we derive numerical simulation of ER variations. Figure 7b shows the comparison between simulation and experimental results during cooling. When *A*→*R* and *A*→*M* occur, the simulation results are in good agreement with the experimental results. When *A*→*R*+*M* occurs, the model reveals a relatively large error because the ER variations represent the mixture effect of R-phase and martensite.

## 6. Self-Sensing Modeling of SMA Actuated AM

To achieve self-sensing function of the SMA-AM, we should establish resistance-length (R-L) relationships of SMA wires. The resistivity-strain *ρ* − *ε* relationship can be established firstly. According to the previous discussion, ER variations of SMA wires depend mainly on the extent of transformation during phase transformation, which can be described by martensite fraction [15]. Besides, the whole resistivity value also varies with applied stresses according to our finding. Thus the ER and martensitic fraction satisfy the following relationships:

(16a) $$\rho_{A↔M}=\rho_{A}^{0}+(\rho_{M}^{0}-\rho_{A}^{0})\xi +\beta_{A,M}\sigma \;\text{without R}‐\text{phase}$$

(16b) $$\{\begin{array}{l} \rho_{A↔R}=\rho_{A}^{0}+(\rho_{R}^{0}-\rho_{A}^{0})\xi_{R}+\beta_{A,R}\sigma \\ \rho_{R↔M}=\rho_{R}^{0}+(\rho_{M}^{0}-\rho_{R}^{0})\xi_{R}+\beta_{R,M}\sigma \end{array}\text{with R}‐\text{phase}$$

where the superscript 0 denotes the ER at free stress. *β_i_* is the stress coefficient of ER, which can be considered as a constant. In addition, the following constitutive model is used in this study [18]:

(17) $$\sigma -\sigma_{0}=E(ɛ-{ɛ}_{0})+\Theta (T-T_{0})+\Omega (\xi -\zeta_{0})$$

where *E* is Young's modulus, Θ is thermal coefficient of expansion, and Ω is the transformation tensor of SMA. Besides, Ω = −*Eε_L_*, and *ε_L_* as a constant is the maximum residual strain. Thus, we can obtain the following ρ − *ε* expression by substituting Equation (16) into Equation (17):

(18) $$\rho =\{\begin{array}{l} K_{M\to A}ɛ+\rho_{A}^{0}+\beta_{A}\sigma \;\text{for}\;M\to A\\ K_{A\to A}ɛ+\rho_{A}^{0}+\beta_{A}\sigma \;\text{for}\;A\to R\\ K_{R\to M}ɛ+\rho_{R}^{0}+\beta_{R}\sigma \;\text{for}\;R\to M \end{array}$$

where $K_{M\to A}=(\rho_{M}^{0}-\rho_{A}^{0})/{ɛ}_{L},K_{A\to R}=(\rho_{R}^{0}-\rho_{A}^{0})/{ɛ}_{L}^{R}$ and $K_{R\to M}=(\rho_{M}^{0}-\rho_{R}^{0})/({ɛ}_{L}-{ɛ}_{L}^{R}),{ɛ}_{L}^{R}$ is maximum residual strain of R-phase transformation, which is generally far smaller than *ε_L_*.

**Figure 9.**
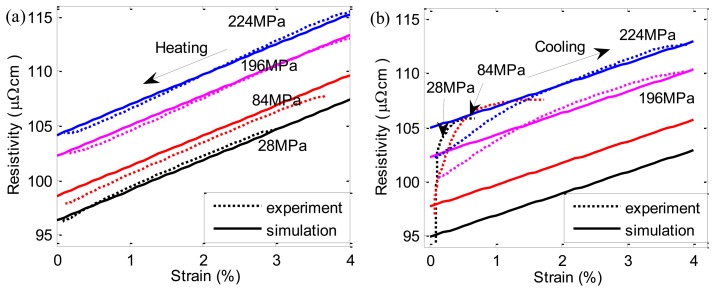
Simulation and experimental results of ER *vs.* strain curves during *M*→*A* (**a**) and *A*→*M* (**b**) transformation at various constant stresses.

The simulation and experimental results of *ρ* − *ε* curves during *M*→*A* and *A*→*M* are shown in Figure 9. During *M*→*A*, the *ρ* − *ε* curves have good linearity. The whole resistivity value changes linearly with the stress and the simulation results agree well with the experimental results. However, during *A*→ *M*, a large error occurs between simulation and experimental results at lower applied stresses. This is due to the appearance of R-phase during cooling. With the increase of the stress, R-phase fraction decreases and the linearity becomes better.

**Figure 10.**
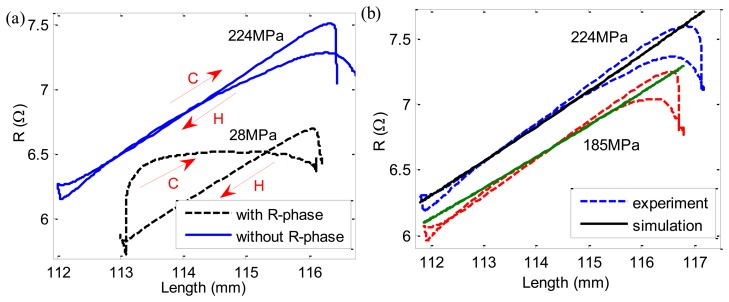
experimental and simulation results of R-L relationships.

The experimental resistance-length (R-L) curves are shown in Figure 10a. When the applied stress is large, R-phase is inhibited and the linearity of R-L curve is quite good and the hysteresis gap of heating and cooling path is also small. However, when the applied stress is small, the resistance-length curve has two different steps with very different slopes during cooling caused by R-phase, which is not convenient for self-sensing application. Here we are only concerned with the case without R-phase. Assuming the initial length of SMA wire is *l*_0_, then:

(19) $$ɛ=\frac{l-l_{0}}{l_{0}}$$

Combining Equations (12), (18) and (19), we can gain the following R-L relationship:

(20) $$\{\begin{array}{cc} R_{h}=\frac{1}{V}(\frac{K_{A}}{l_{0}}l^{3}+H_{A}l^{2}) & \;\text{for}\;M\to A\\ R_{c}=\frac{1}{V}(\frac{K_{M}}{l_{0}}l^{3}+H_{M}l^{2}) & \;\text{for}\;A\to M \end{array}$$

where $H_{A}=\beta_{A}\sigma +\rho_{A_{f}}^{0}-K_{A}$ and $H_{M}=\beta_{M}\sigma +\rho_{M_{s}}^{0}-K_{M}$. The values *R_h_* and *R_c_* are the resistance on the heating path and cooling path respectively. As the heating and cooling paths are very close to each other, we average the two paths as follows:

(21) $$R=\frac{R_{h}+R_{c}}{2}$$

Combining Equations (19) and (20), we can gain simulation results of R-L relationships under different applied stresses, which agree well with experimental results as shown in Figure 10b.

**Figure 11.**
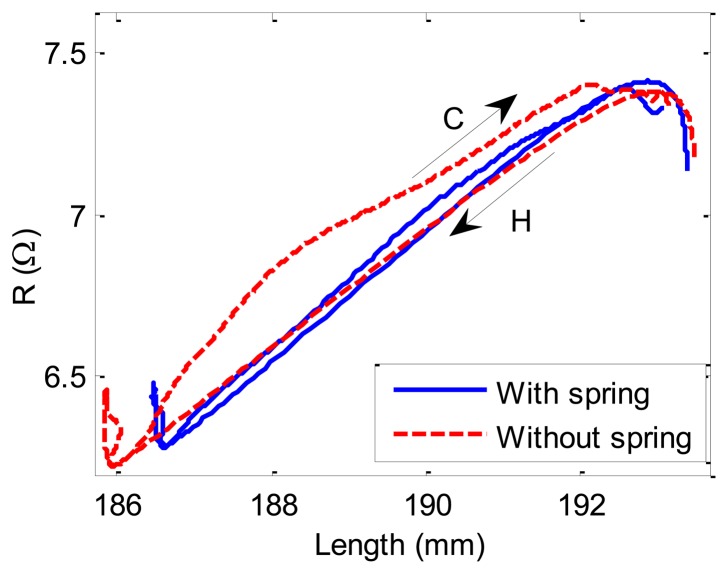
Experimental results of R-L relationships under constant (without spring) and variable (with spring) loads.

The above studies were performed under constant stress. In practice, the SMA-AM generally contracts against variable stress. In order to demonstrate the correctness of the self-sensing model under variable stress conditions, the same experiments are performed by adding a bias spring with stiffness of 143 N/m under the constant load of 56 MPa as shown in Figure 4. The R-L curves with and without bias spring are shown in Figure 11. The differences between the two curves are minimal during heating path (H arrow) and they have the same slope. However, during cooling process (C arrow), there is a big difference between the two curves, which is caused by SMA R-phase. Therefore, as long as the R-phase is inhibited, the two curve can be coincide well with each other. Thus, the self-sensing model (Equations (20) and (21)) can predict the R-L relationships both under contant and variable stresses, and the self-sensing can be achieved for the SMA-AM.

It should be noted that, the above self-sensing model is developed based on natural cooling. Different cooling methods (such as natural cooling and forced cooling) have different heat transfer rate, as shown in Subsection 5.1, which is represented by convection coefficient *h_c_*. However, the self-sensing is not affected by the cooling method. As it is developed from ER variations and the ER depends mainly on martensite fraction during transformation, which is independent of heat transfer rate [17].

## 7. Application to Active Ankle-Foot Orthosis (AAFO)

**Figure 12.**
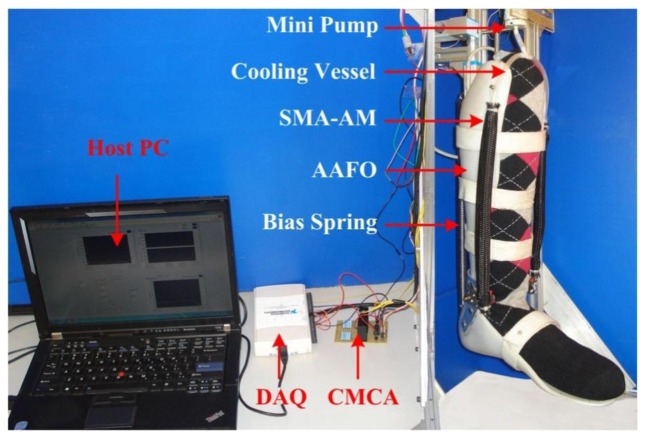
Artificial skeletal muscle actuated 1-DOF robotic ankle-foot.

To further demonstrate the self-sensing capability of the SMA-AM, we build an Active Ankle-Foot Orthosis (AAFO) actuated and sensed by the SMA-AM and with a bias spring with stiffness of 1,144 N/m as shown in Figure 12. The SMA-AM can provide a maximum contractile force of 230 N. The maximum torque of the AAFO can be determined as 10.9 Nm. It should be noted that different patients require different AAFO assist torque, which can be achieved by adding or reducing the amount of parallel SMA wires. The bias spring serves two purposes: to provide enough restoring force for the AM and to simulate variable load condition. The orthosis combines two light-weight thermoplastic shells with articulated joints, which can provide supplemental torque assistance for weak anterior muscles of the lower leg during dorsiflexion. Besides, stable and continuous airflow is applied to cool the AMs through the cooling vessel provided by a mini pump. The current heating and data processing methods are similar to the previous experiments. Before experiments, an initial pre-transformation stress of σ = 112 MPa, is applied on the SMA-AM to inhibit the appearance of R-phase.

**Figure 13.**
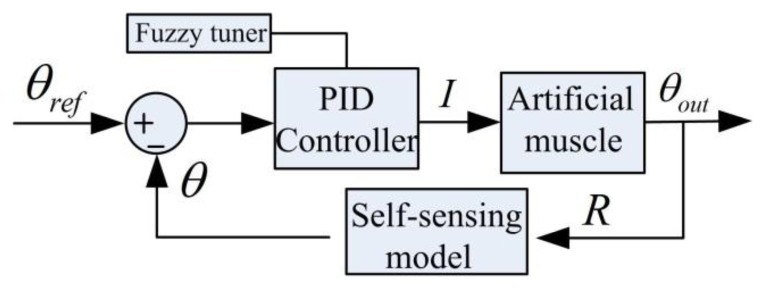
Fuzzy PID control diagram.

A fuzzy-tuned PID controller is implemented in the Labview environment to control the AAFO. The PID paremeters are tuned between *K_P_* = 0.1 − 0.5, *K_I_* = 0.01 − 0.06 and *K_D_* = 0.001 − 0.002 by using MAX-MIN fuzzy reasoning method similar to [11]. The control diagram is shown in Figure 13.

When the AAFO angle changes, the SMA-AM length can be determined according to Equation (22):

(22) $$l-l_{0}=\frac{a}{2}\sin(\theta -\theta_{0})$$

where *l* is the SMA-AM length, *θ* is the angular position and *a*/2 is the moment arm of the AAFO. The subscript 0 denotes the initial length and angle value.

**Figure 14.**
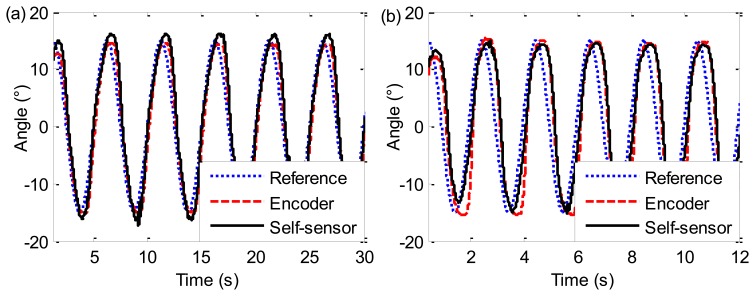
Sinusoidal tracking response at 0.2 Hz (**a**) and 0.5 Hz (**b**).

The resistance of the SMA wire is measured as the feedback signal and then converted to the ankle angle combining the self-sensing model (Equations (20) and (21)) with Equation (22). Furthermore, the actual angle is also used for the feedback control as a comparison. Figure 14a shows the results of sinusoidal tracking response experiments using the self-sensor and encoder respectively at 0.2 Hz. Note that, the “Reference” angle *θ_ref_* is the desired sinusoidal curve produced by host PC. The “Encoder” angle is the actual curve sensed by an encoder, and the “Self-sensor” angle is the actual curve sensed by the self-sensing model. The actual angle with the two feedback methods can accurately follow the reference angle, which demonstrates the validity of the self-sensing method. When the sinusoidal frequency increases to 0.5 Hz as shown in Figure 14b, a small phase lag appears between the actual angle and the reference angle. But the two actual angle curves coincide well with each other, because both resistance and length change of SMA wires are closely related to SMA transformation, which further demonstrates the validity of the self-sensing model.

## 8. Conclusions

This paper proposes a self-sensing model to achieve the integrated muscle-like functions of actuating and self-sensing for the designed SMA-AM based on a deep investigation of SMA electrical resistivity (ER). An ER transformation kinetics model is firstly established based on SMA DSC tests. Then a series of thermal-electrical-mechanical experiments are carried out to verify the validity of the ER model, whereby the SMA-AM self-sensing function is well established under different stress conditions. A novel AAFO actuated and sensed by the SMA-AM is built to further demonstrate the self-sensing capability of the SMA-AM. It is shown that the AAFO can accurately follow the desired angle value within SMA bandwidth, which is also robust to external stress variations.

## Figures and Tables

**Table 1.** Experimental material parameters of SMA wires.

	**Martensite**	**R-Phase**	**Austenite**
TCR: *α_i_* (μΩcm·°C^−1^)	0.08	−0.05	0.05
SCR: *β_i_* (μΩcm·°C^−1^)	0.05	0.04	0.04
Stress on temp.: C*_i_* (MPa·°C^−1^)	3.68	-	6.06
ER at zero stress and *T*_0_*_i_*: $p_{0i}^{0}$ (μΩcm)	100.5	104.2	95.2
*T*_0_*_i_* (°C)	34	58	79

**Table 2.** DSC parameters of SMA wires.

	*M*→*A*	*A*→*R*	*R*→*M*
Start trans. temp. at zero stress (°C)	73	-	44
Peak trans. temp. at zero stress (°C)	76	58	39
Finish trans. temp. at zero stress (°C)	79	-	34
Latent heat: *Q_i_* (J/g)	24.5	0	11.2
Max. recov. strain (%)	4.8	0.8	4

## References

[1] Zhang J.J., Zhu J.Y. 4M-Model Based Bionic Design of Artificial Skeletal Muscle Actuated by SMA.

[2] Zhang J.J., Yin Y.H. (2012). SMA-based bionic integration design of self-sensor-actuator-structure for artificial skeletal muscle. Sens. Actuatators A Phys..

[3] Zhang J.J., Yin Y.H., Zhu J.Y. (2013). Sigmoid-based hysteresis modeling and high-speed tracking control of SMA-artificial muscle. Sens. Actuators A Phys..

[4] Yin Y.H., Hu H., Xia Y.C. (2004). Active tracking of unknown surface using force sensing and control technique for robot. Sens. Actuatators A Phys..

[5] Yin Y.H., Fan Y.J., Xu L.D. (2012). EMG and EPP-integrated human-machine interface between the paralyzed and rehabilitation exoskeleton. IEEE Trans. Inf. Technol. B..

[6] Fan Y.J., Guo Z., Yin Y.H. (2011). Semg-based neuro-fuzzy controller for a parallel ankle exoskeleton with proprioception. Int. J. Robot. Autom..

[7] Klute G.K., Czerniecki J.M., Hannaford B. (2002). Artificial muscles: Actuators for biorobotic systems. Int. J. Robot. Res..

[8] Lan C.C., Yang Y.N. (2009). A computational design method for a shape memory alloy wire actuated compliant finger. J. Mech. Des..

[9] Zhong Z.W., Yeong C.K. (2006). Development of a gripper using SMA wire. Sens. Actuatators A Phys..

[10] Esfahani E.T., Elahinia M.H. (2007). Stable walking pattern for an SMA-actuated biped. IEEE ASME Trans. Mech..

[11] Lan C.C., Fan C.H. (2010). An accurate self-sensing method for the control of shape memory alloy actuated flexures. Sens. Actuatators A Phys..

[12] Lan C.C., Lin C.M., Fan C.H. (2011). A self-sensing microgripper module with wide handling ranges. IEEE ASME Trans. Mech..

[13] Cui D., Song G.B., Li H.N. (2010). Modeling of the electrical resistance of shape memory alloy wires. Smart Mater. Struct..

[14] Novak V., Sittner P., Dayananda G.N., Braz-Fernandes F.M., Mahesh K.K. (2008). Electric resistance variation of NiTi shape memory alloy wires in thermomechanical tests: Experiments and simulation. Mater. Sci. Eng. A Struct..

[15] Lagoudas D.C. (2008). Shape Memory Alloys: Modeling and Engineering Applications.

[16] He Z., Gall K.R., Brinson L.C. (2006). Use of electrical resistance testing to redefine the transformation kinetics and phase diagram for shape-memory alloys. Metall. Mater. Trans. A..

[17] Otsuka K., Wayman C.M. (1999). Shape Memory Materials.

[18] Brinson L. (1993). One-dimensional constitutive behavior of shape memory alloys: Thermomechanical derivation with non-constant material functions and redefined martensite internal variable. J. Intell. Mater. Syst. Struct..

[19] Pozzi M., Airoldi G. (1999). The electrical transport properties of shape memory alloys. Mater. Sci. Eng. A Struct..

[20] Faulkner M., Amalraj J., Bhattacharyya A. (2000). Experimental determination of thermal and electrical properties of Ni-Ti shape memory wires. Smart Mater. Struct..

[21] Bhattacharyya A., Sweeney L., Faulkner M. (2002). Experimental characterization of free convection during thermal phase transformations in shape memory alloy wires. Smart Mater. Struct..

[22] Churchill C.B., Shaw J.A. (2008). Shakedown response of conditioned shape memory alloy wire. Proc. SPIE.

